# Autologous Rib Grafts for Sternal Reconstruction After Excision of a Chondrosarcoma

**DOI:** 10.1016/j.atssr.2024.03.005

**Published:** 2024-04-02

**Authors:** Ryusuke Sumiya, Mariko Fukui, Yukio Watanabe, Takeshi Matsunaga, Aritoshi Hattori, Tsuyoshi Saito, Kazuya Takamochi, Kenji Suzuki

**Affiliations:** 1 Department of General Thoracic Surgery, Juntendo University School of Medicine, Tokyo, Japan; 2 Department of Human Pathology, Juntendo University School of Medicine, Tokyo, Japan

## Abstract

Sternal chondrosarcoma is a rare malignant condition. Although surgical resection is crucial, the reconstruction of sternal defects is challenging. A 64-year-old male patient with a history of 2 separate sternal tumor resections received a diagnosis of sternal chondrosarcoma recurrence. Because the tumor caused bone destruction and invasion into the sternum, the sternum and tumor were resected. Sternal reconstruction with autologous ribs was performed. Although the tumor was diagnosed as a recurrent chondrosarcoma, the patient had a long disease-free survival postoperatively. Local control is important in chondrosarcomas, and autogenous rib grafts may be an option for patients with sternal defects.

Sternal chondrosarcomas are extremely rare. Chest wall chondrosarcomas comprise 15% of all chondrosarcomas, of which sternal chondrosarcomas account for 2%.^1^ Although surgical resection is crucial to the outcome of chondrosarcomas, insufficient resection can cause local recurrence.^2^ Reconstruction of sternal defects is challenging because it is fraught with the risk of flail chest, paradoxical respiration, kyphosis, respiratory dysfunction, and physical dysfunction.^3^ Here we present a rare case of a patient with 8-year disease-free survival after tumor and sternal resection and autologous rib reconstruction for recurrent chondrosarcoma of the sternum.

A 64-year-old ex-smoking male patient with a history of sternal tumors presented with a mass on the anterior sternal surface. He had undergone tumor resection at the ages of 46 and 49 years. The tumor was pathologically diagnosed as a chondroma at the first operation but was revised as a chondrosarcoma at the second surgery. Under the diagnosis of malignancy, he underwent radiation therapy (50 gray/25 fraction) after tumor resection. However, he was aware of pain and bulging of the anterior sternal surface at the age of 61 years, and he was referred to our hospital (Juntendo University School of Medicine, Tokyo, Japan) for further examination and treatment at the age of 64 years. He had no medical history except for the sternal tumors, and there were no abnormalities in his tumor markers or chest radiography. Chest computed tomography revealed an 83 × 45 mm sternal mass with a contrast-enhanced edge (Figure 1). T1- and T2-weighted magnetic resonance imaging revealed an anterior mediastinal tumor as low-intensity and high-intensity areas, respectively. There was a maximum standard uptake value of 2.1 to 2.6 with fluorodeoxyglucose and 2-[fluorine-18] fluoro-1-deoxy-D-glucose accumulation on positron emission tomography combined with computed tomography. He received a diagnosis of recurrent sternal chondrosarcoma, and surgical resection was planned.

**Figure 1:**
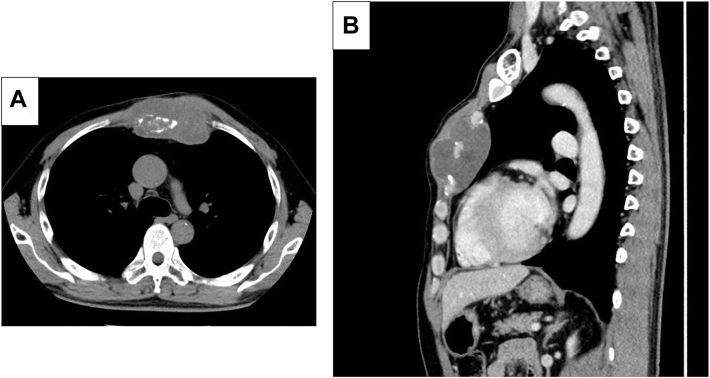
Radiologic findings. (A) Axial and (B) sagittal computed tomographic image showing an 83 × 45 mm sternal mass with a contrast-enhanced edge.

Operation was performed using general and epidural anesthesia with the patient in the supine position. Because the tumor caused bone destruction and sternal invasion, the connections of the bilateral ribs and the sternum were dissected, and the previous skin incision, the sternal body, and the tumor were resected en bloc. There was a broad, full-thickness chest wall defect, and 3 layers (pleura wall, osteothorax, and body surface) were reconstructed. First, internal fixation with a 2-mm Gore-Tex sheet (W.L. Gore & Associates) was performed (Figure 2A). Second, the seventh left (15-cm) and sixth right (12-cm) ribs were harvested to reconstruct the osteothorax. Sternal reconstruction with autologous ribs was performed using the right sixth rib between the right and left second ribs and the left seventh rib between the right third and left fourth ribs (Figure 2B). Finally, the body surface was reconstructed using a pectoralis major musculocutaneous flap (Figure 2C). The postoperative course was uneventful, and the patient was discharged 10 days postoperatively under sufficient pain control through the use of oral nonsteroidal antiinflammatory drugs. The tumor was diagnosed as a chondrosarcoma grade 2 recurrence. Follow-up chest computed tomography 8 years postoperatively did not show evidence of tumor recurrence. Follow-up chest computed tomography revealed that autologous rib grafts were sequestrated 3 years postoperatively. However, the chest wall was thickened, and the patient remained without kyphosis, respiratory failure, or exercise restrictions (Figure 3).

**Figure 2:**
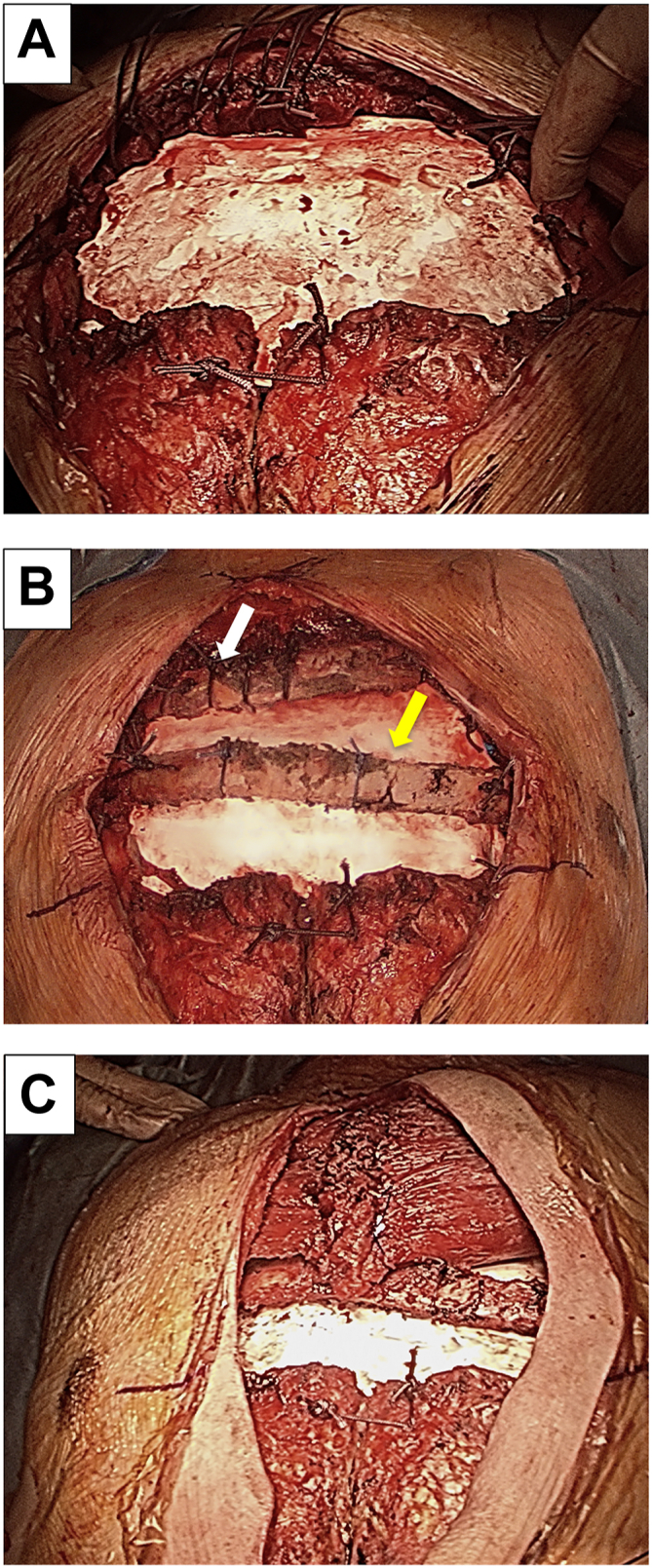
Intraoperative findings. (A) Internal fixation with a 2-mm Gore-Tex sheet (W.L. Gore & Associates). (B) Sternal reconstruction with autologous ribs. The white arrow indicates the right sixth rib between the right and left second ribs, and the yellow arrow indicates the left seventh rib between the right third and left fourth ribs. (C) Body surface reconstruction using a pectoral major musculocutaneous flap.

**Figure 3:**
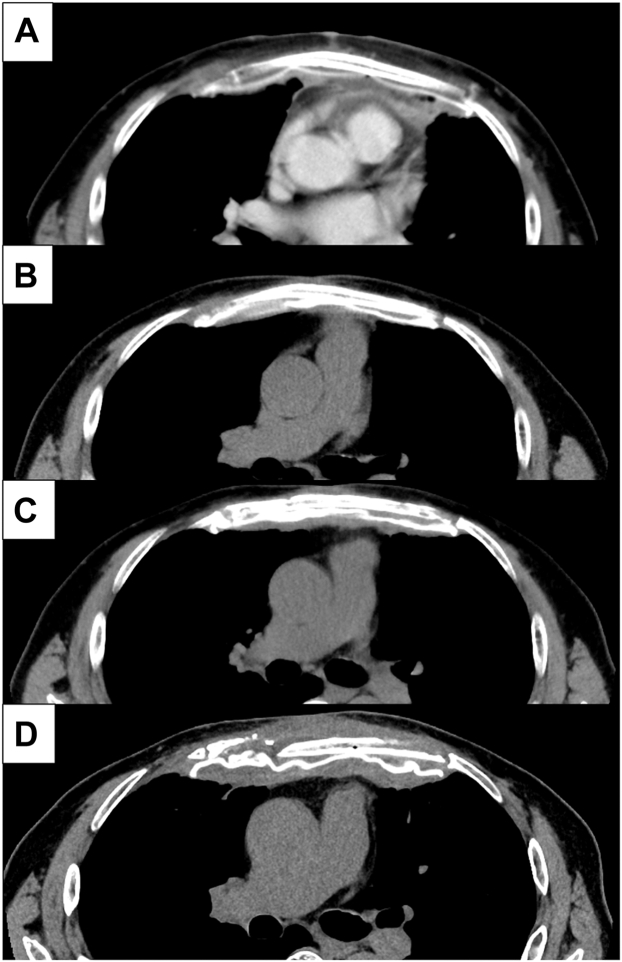
Radiologic findings (A) 1 month, (B) 1 year, (C) 3 years, and (D) 8 years postoperatively.

## Comment

The incidence of chondrosarcomas arising from osteochondromas is 0.4% to 2% in patients with solitary osteochondromas.^4^ Surgery is crucial for the outcome of chondrosarcomas given their insensitivity to radiotherapy or chemotherapy. Although chondrosarcoma has a better survival (5-year overall survival rates, 64%-92%^5^), Kim and colleagues^6^ reported that patients with local chondrosarcoma recurrence showed poor survival rates (5-year overall survival rate, 58.6%). Kim and colleagues^6^ also reported poor survival risks, such as age >50 years and local recurrence occurring within 1 year after the primary surgery. Furthermore, the recurrence rate after local resection for primary chest wall chondrosarcomas has been reported to be 33% to 50%.^2^^,^^7^ and it may be even higher for primary sternal chondrosarcomas because of the reconstruction difficulty. The most important factor affecting survival is whether the primary neoplasm is completely controlled. In the present case, the patient experienced local recurrence twice because inadequate surgical resection was performed during the first and second operations.

We encountered difficulties because a large excision was performed during the initial procedure to stop local recurrence; thus, rigid and nonrigid chest wall reconstructions were required. Chest wall reconstruction eliminated the potential dead space, permitted recreation of a rigid thorax, minimized the visible deformity, and protected the intrathoracic organs. Fulfilling these criteria should minimize the infection risk, maximize the patient’s ability to receive adjuvant radiation, and maintain cosmesis and pulmonary mechanics. Large chest wall resections can be associated with significant morbidities, such as flail chest and paradoxical respiration, as well as long-term morbidity affecting patients’ abilities to perform activities of daily living, such as kyphosis, respiratory dysfunction, and physical dysfunction; therefore, adequate reconstruction is critical for patient outcomes.^3^ The defect location, size, and patient background play major roles in selecting the reconstruction method. Although titanium plates are widely used in the rigid reconstruction of sternal defects, autogenous rib grafts have many advantages, such as biocompatibility, stabilization, backscatter at radiation, and a lack of risk of diseases associated with prosthetic materials.^8^ Moreover, these grafts are easier to obtain and have lower economic costs than prosthetic materials. In our case, autogenous rib grafting was successfully used to reconstruct the sternal defects after tumor and partial sternal resection. Although long-term observation revealed that the rib grafts were sequestered, chest wall and surrounding tissue thickening allowed no further complications. There have been no reports on long-term follow-up after sternal reconstruction; therefore, additional studies are needed. We believe that autogenous rib grafts could be a viable alternative for patients with sternal defects resulting from infections, tumors, or trauma that require grafting for sternal stabilization.

We experienced a patient with a good clinical outcome and long-term prognosis after surgical tumor resection, sternal resection, and autologous rib reconstruction for recurrent sternal chondrosarcoma. Local control is very important in chondrosarcomas, and it is important to consider reconstruction with a rigid material if a large sternal excision is required.
